# Mode of Preserving Nitrate of Silver

**Published:** 1842-12

**Authors:** 


*Mode of Preserving Nitrate of Silver.*—M. Dumeril has for a long while employed a very simple process for preserving the nitrate of silver from the injurious effects of exposure to the air, when run into sticks. It consists in merely coating the caustic with engraver's sealing-wax, which contains a large quantity of shellac. This wax adheres very well, and forms a strong and smooth varnish, as it were, which remains unaffected by the atmosphere. Thus protected the nitrate no longer stains the fingers, injures the caustic-case, nor is in any way changed by the moisture in the air, possesses a greater degree of solidity, and, at the same time, the process is of exceeding service in practice, inasmuch as when wanted for use a small part only of the caustic need be uncovered by means of a penknife, so that its application can be restricted to the part where it is required. This is of peculiar utility in ulceration of the throat, aphthæ, fissures, &c.—*Prov. Med. Journ.* July 2, from *Bull. de Thérap.*

